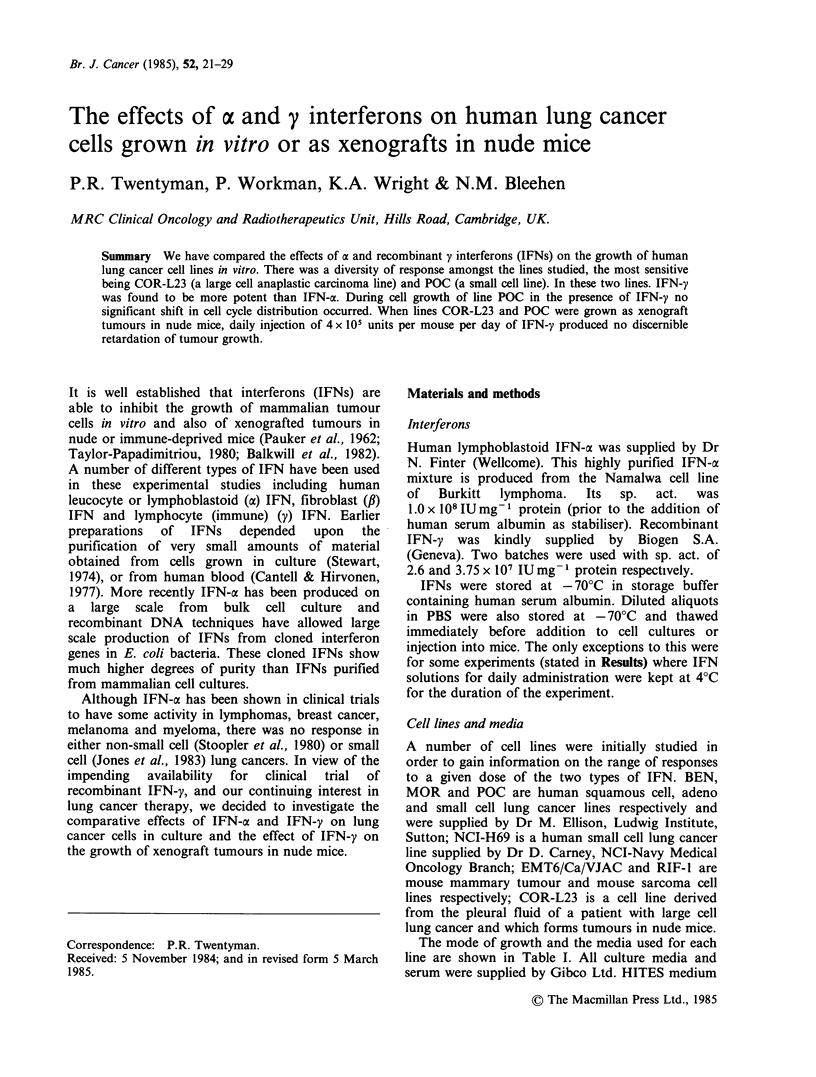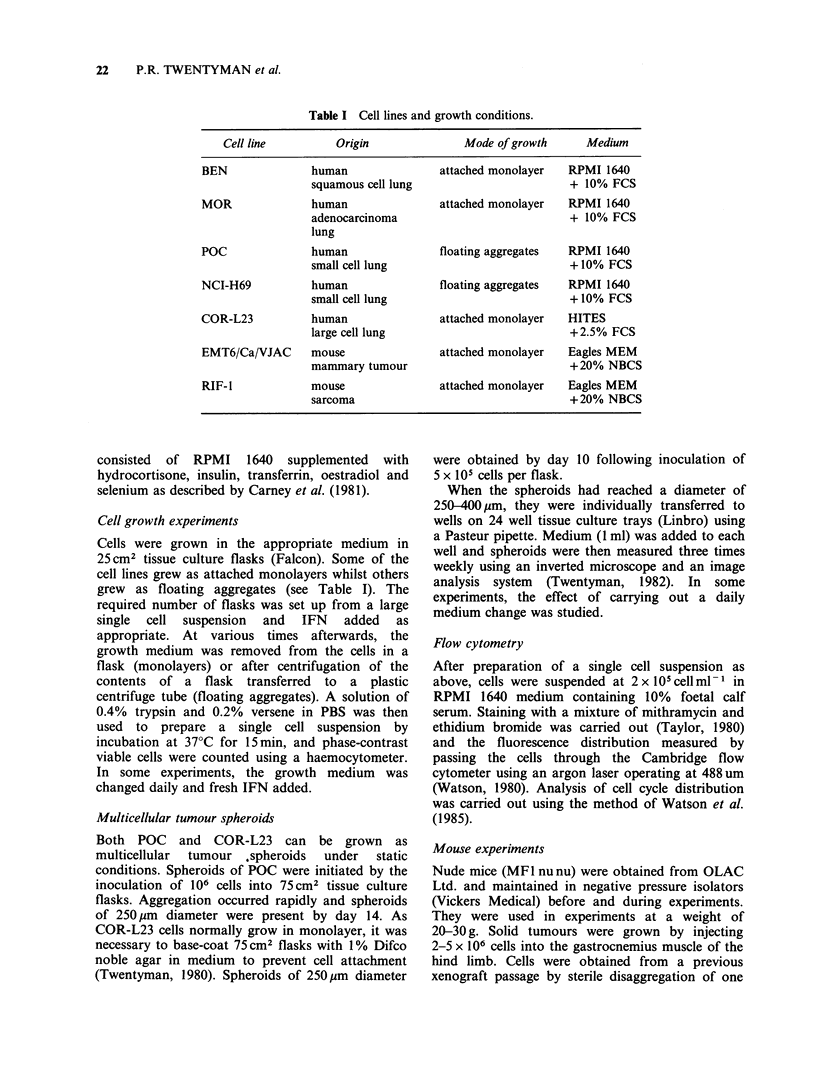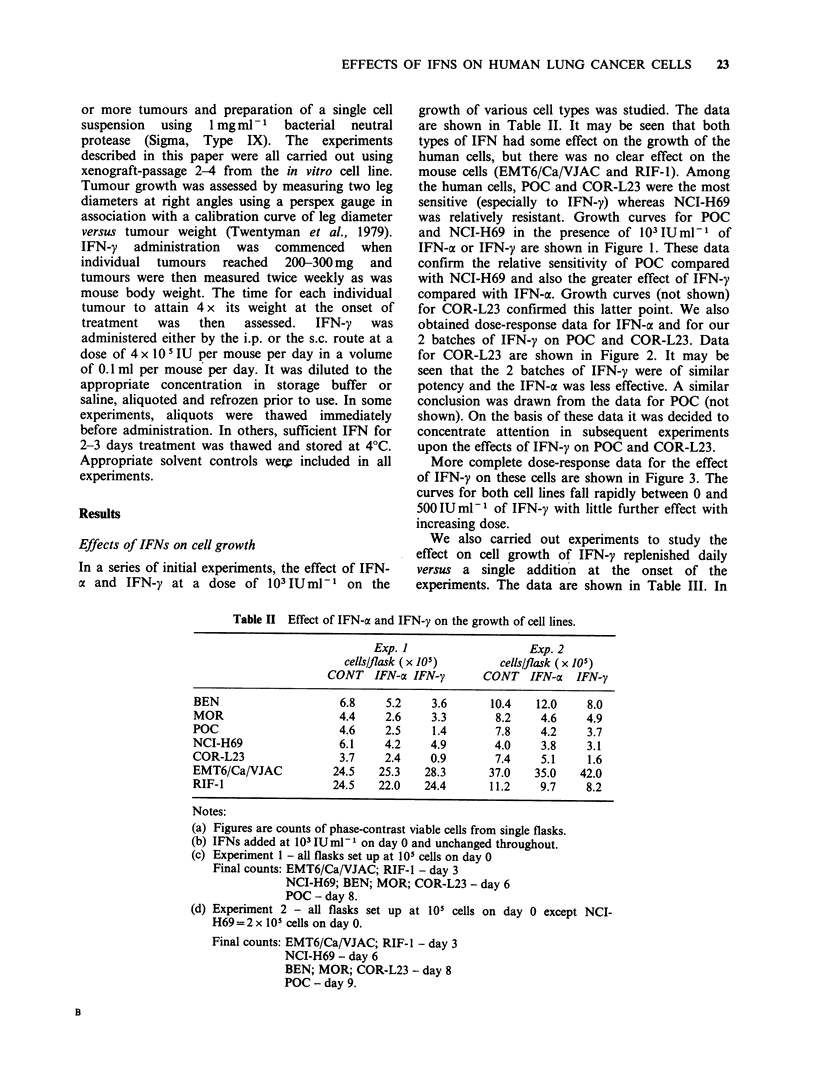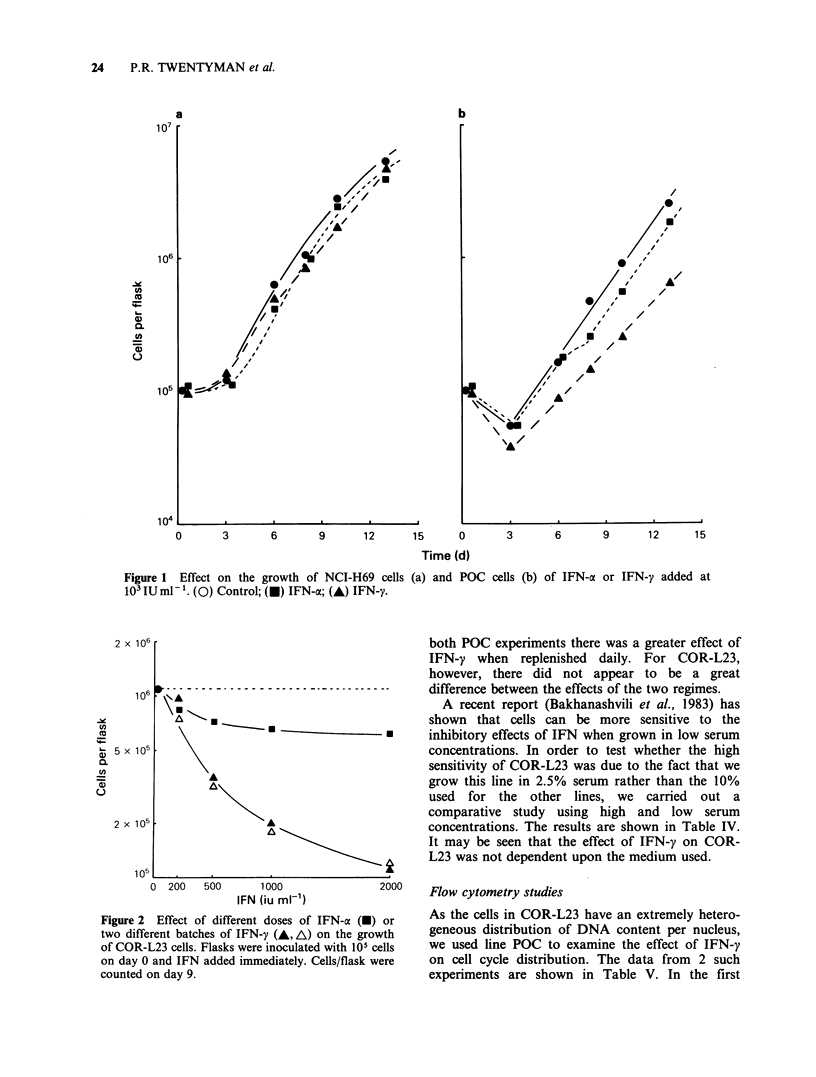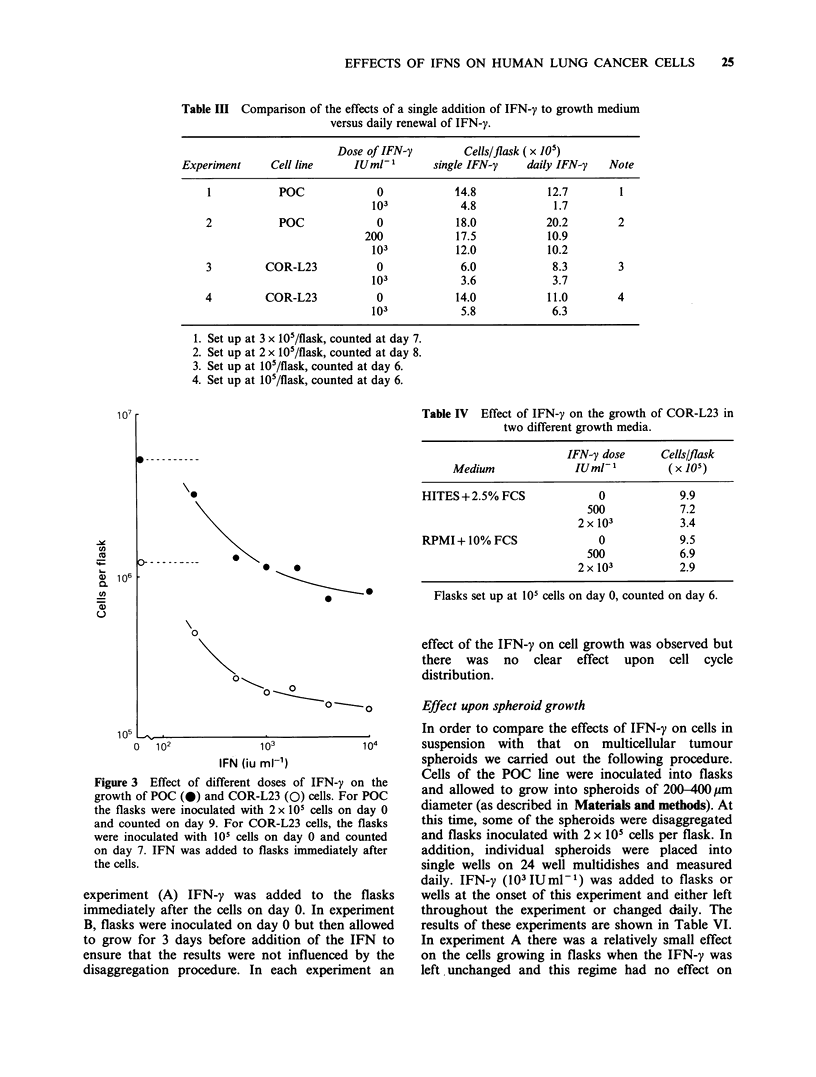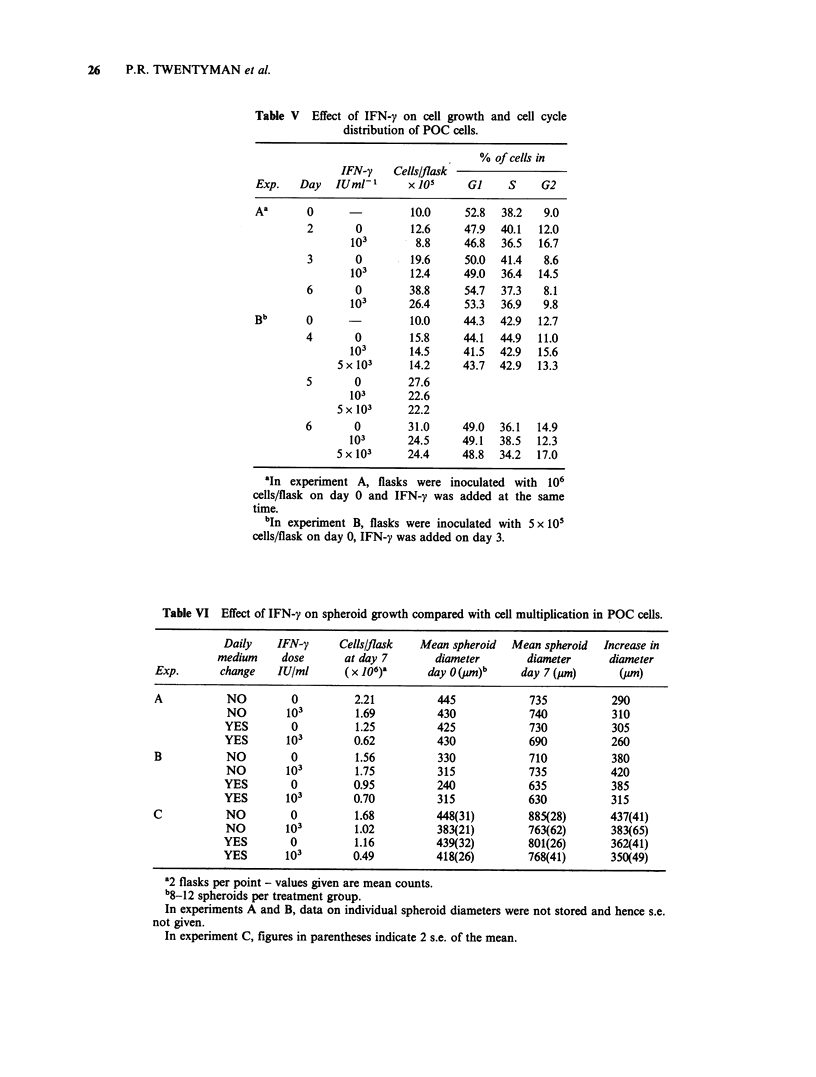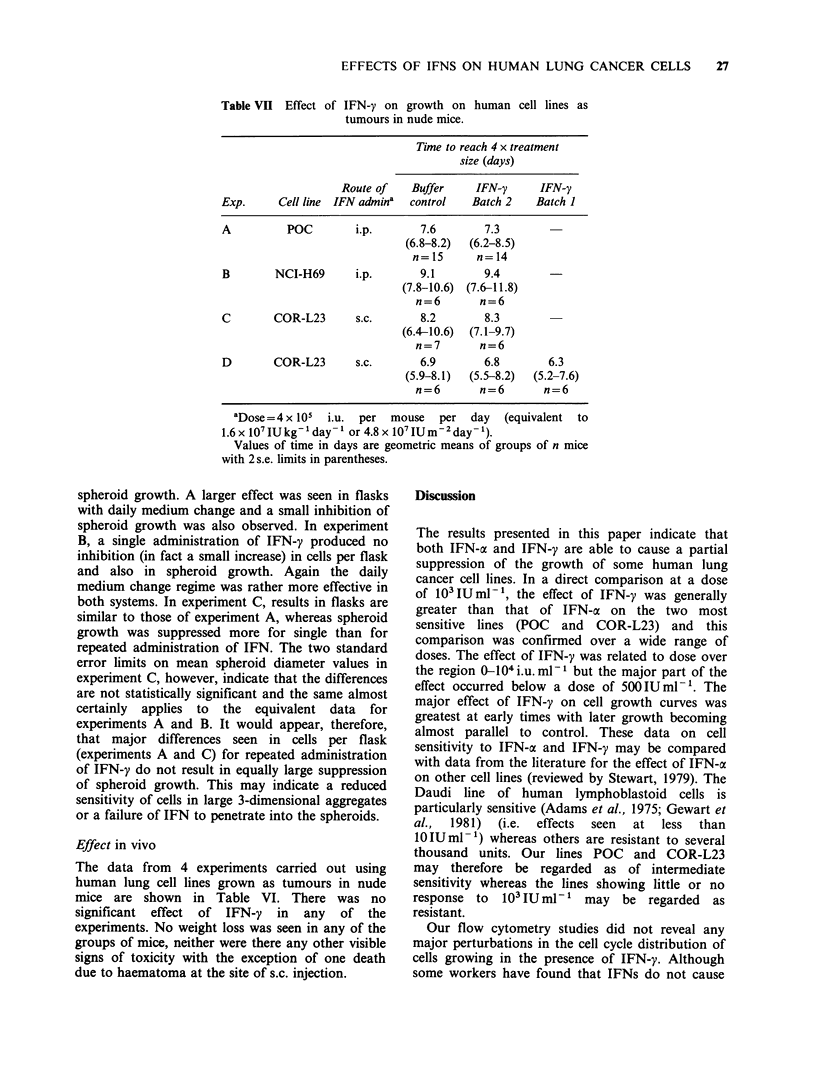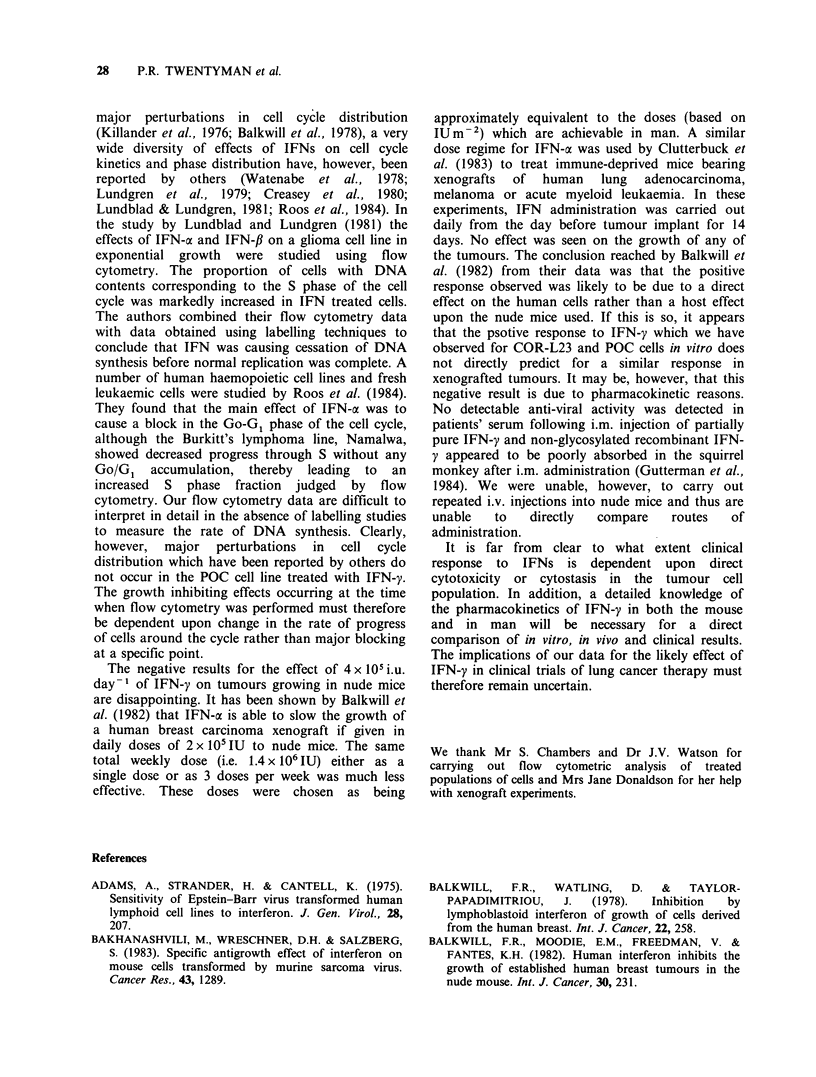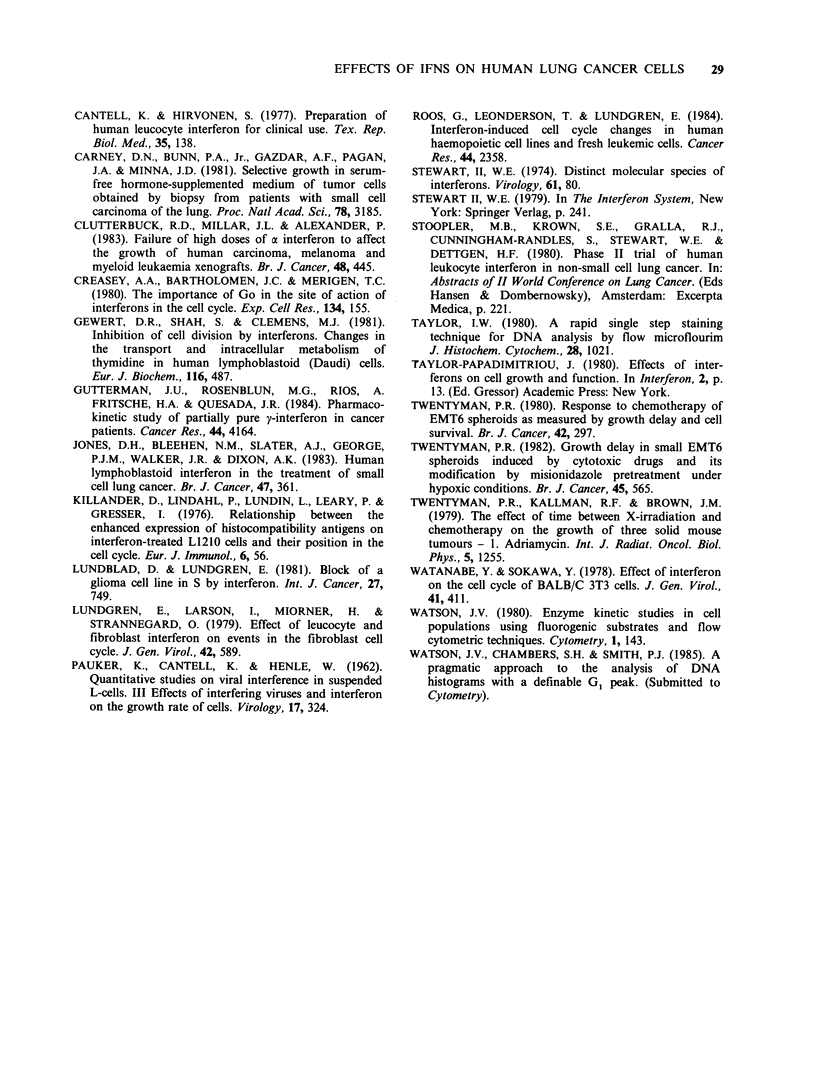# The effects of alpha and gamma interferons on human lung cancer cells grown in vitro or as xenografts in nude mice.

**DOI:** 10.1038/bjc.1985.143

**Published:** 1985-07

**Authors:** P. R. Twentyman, P. Workman, K. A. Wright, N. M. Bleehen

## Abstract

We have compared the effects of alpha and recombinant gamma interferons (IFNs) on the growth of human lung cancer cell lines in vitro. There was a diversity of response amongst the lines studied, the most sensitive being COR-L23 (a large cell anaplastic carcinoma line) and POC (a small cell line). In these two lines, IFN-gamma was found to be more potent than IFN-alpha. During cell growth of line POC in the presence of IFN-gamma no significant shift in cell cycle distribution occurred. When lines COR-L23 and POC were grown as xenograft tumours in nude mice, daily injection of 4 X 10(5) units per mouse per day of IFN-gamma produced no discernible retardation of tumour growth.


*Br. J. Cancer* (1985), **52**, 21–29

### The effects of α and γ interferons on human lung cancer cells grown *in vitro* or as xenografts in nude mice

P.R. Twentyman, P. Workman, K.A. Wright & N.M. Bleehen

*MRC Clinical Oncology and Radiotherapeutics Unit, Hills Road, Cambridge, UK.*

**Summary** We have compared the effects of α and recombinant γ interferons (IFNs) on the growth of human lung cancer cell lines *in vitro*. There was a diversity of response amongst the lines studied, the most sensitive being COR-L23 (a large cell anaplastic carcinoma line) and POC (a small cell line). In these two lines. IFN-γ was found to be more potent than IFN-α. During cell growth of line POC in the presence of IFN-γ no significant shift in cell cycle distribution occurred. When lines COR-L23 and POC were grown as xenograft tumours in nude mice, daily injection of $4\times10^5$ units per mouse per day of IFN-γ produced no discernible retardation of tumour growth.

It is well established that interferons (IFNs) are able to inhibit the growth of mammalian tumour cells *in vitro* and also of xenografted tumours in nude or immune-deprived mice (Pauker *et al.*, 1962; Taylor-Papadimitriou, 1980; Balkwill *et al.*, 1982). A number of different types of IFN have been used in these experimental studies including human leucocyte or lymphoblastoid (α) IFN, fibroblast (β) IFN and lymphocyte (immune) (γ) IFN. Earlier preparations of IFNs depended upon the purification of very small amounts of material obtained from cells grown in culture (Stewart, 1974), or from human blood (Cantell & Hirvonen, 1977). More recently IFN-α has been produced on a large scale from bulk cell culture and recombinant DNA techniques have allowed large scale production of IFNs from cloned interferon genes in *E. coli* bacteria. These cloned IFNs show much higher degrees of purity than IFNs purified from mammalian cell cultures.

Although IFN-α has been shown in clinical trials to have some activity in lymphomas, breast cancer, melanoma and myeloma, there was no response in either non-small cell (Stoopler *et al.*, 1980) or small cell (Jones *et al.*, 1983) lung cancers. In view of the impending availability for clinical trial of recombinant IFN-γ, and our continuing interest in lung cancer therapy, we decided to investigate the comparative effects of IFN-α and IFN-γ on lung cancer cells in culture and the effect of IFN-γ on the growth of xenograft tumours in nude mice.

Correspondence: P.R. Twentyman.
Received: 5 November 1984; and in revised form 5 March 1985.

### Materials and methods

### *Interferons*

Human lymphoblastoid IFN-α was supplied by Dr N. Finter (Wellcome). This highly purified IFN-α mixture is produced from the Namalwa cell line of Burkitt lymphoma. Its sp. act. was $1.0\times10^8$ IU mg$^{-1}$ protein (prior to the addition of human serum albumin as stabiliser). Recombinant IFN-γ was kindly supplied by Biogen S.A. (Geneva). Two batches were used with sp. act. of 2.6 and $3.75\times10^7$ IU mg$^{-1}$ protein respectively.

IFNs were stored at −70°C in storage buffer containing human serum albumin. Diluted aliquots in PBS were also stored at −70°C and thawed immediately before addition to cell cultures or injection into mice. The only exceptions to this were for some experiments (stated in **Results**) where IFN solutions for daily administration were kept at 4°C for the duration of the experiment.

### *Cell lines and media*

A number of cell lines were initially studied in order to gain information on the range of responses to a given dose of the two types of IFN. BEN, MOR and POC are human squamous cell, adeno and small cell lung cancer lines respectively and were supplied by Dr M. Ellison, Ludwig Institute, Sutton; NCI-H69 is a human small cell lung cancer line supplied by Dr D. Carney, NCI-Navy Medical Oncology Branch; EMT6/Ca/VJAC and RIF-1 are mouse mammary tumour and mouse sarcoma cell lines respectively; COR-L23 is a cell line derived from the pleural fluid of a patient with large cell lung cancer and which forms tumours in nude mice.

The mode of growth and the media used for each line are shown in Table I. All culture media and serum were supplied by Gibco Ltd. HITES medium

© The Macmillan Press Ltd., 1985

**Table I** Cell lines and growth conditions.

| *Cell line* | *Origin* | *Mode of growth* | *Medium* |
|---|---|---|---|
| BEN | human squamous cell lung | attached monolayer | RPMI 1640 + 10% FCS |
| MOR | human adenocarcinoma lung | attached monolayer | RPMI 1640 + 10% FCS |
| POC | human small cell lung | floating aggregates | RPMI 1640 +10% FCS |
| NCI-H69 | human small cell lung | floating aggregates | RPMI 1640 +10% FCS |
| COR-L23 | human large cell lung | attached monolayer | HITES +2.5% FCS |
| EMT6/Ca/VJAC | mouse mammary tumour | attached monolayer | Eagles MEM +20% NBCS |
| RIF-1 | mouse sarcoma | attached monolayer | Eagles MEM +20% NBCS |

consisted of RPMI 1640 supplemented with hydrocortisone, insulin, transferrin, oestradiol and selenium as described by Carney *et al.* (1981).

### *Cell growth experiments*

Cells were grown in the appropriate medium in 25 cm$^2$ tissue culture flasks (Falcon). Some of the cell lines grew as attached monolayers whilst others grew as floating aggregates (see Table I). The required number of flasks was set up from a large single cell suspension and IFN added as appropriate. At various times afterwards, the growth medium was removed from the cells in a flask (monolayers) or after centrifugation of the contents of a flask transferred to a plastic centrifuge tube (floating aggregates). A solution of 0.4% trypsin and 0.2% versene in PBS was then used to prepare a single cell suspension by incubation at 37°C for 15 min, and phase-contrast viable cells were counted using a haemocytometer. In some experiments, the growth medium was changed daily and fresh IFN added.

### *Multicellular tumour spheroids*

Both POC and COR-L23 can be grown as multicellular tumour ,spheroids under static conditions. Spheroids of POC were initiated by the inoculation of $10^6$ cells into 75 cm$^2$ tissue culture flasks. Aggregation occurred rapidly and spheroids of 250 $\mu$m diameter were present by day 14. As COR-L23 cells normally grow in monolayer, it was necessary to base-coat 75 cm$^2$ flasks with 1% Difco noble agar in medium to prevent cell attachment (Twentyman, 1980). Spheroids of 250 $\mu$m diameter were obtained by day 10 following inoculation of $5 \times 10^5$ cells per flask.

When the spheroids had reached a diameter of 250–400 $\mu$m, they were individually transferred to wells on 24 well tissue culture trays (Linbro) using a Pasteur pipette. Medium (1 ml) was added to each well and spheroids were then measured three times weekly using an inverted microscope and an image analysis system (Twentyman, 1982). In some experiments, the effect of carrying out a daily medium change was studied.

### *Flow cytometry*

After preparation of a single cell suspension as above, cells were suspended at $2 \times 10^5$ cell ml$^{-1}$ in RPMI 1640 medium containing 10% foetal calf serum. Staining with a mixture of mithramycin and ethidium bromide was carried out (Taylor, 1980) and the fluorescence distribution measured by passing the cells through the Cambridge flow cytometer using an argon laser operating at 488 um (Watson, 1980). Analysis of cell cycle distribution was carried out using the method of Watson *et al.* (1985).

### *Mouse experiments*

Nude mice (MF1 nu nu) were obtained from OLAC Ltd. and maintained in negative pressure isolators (Vickers Medical) before and during experiments. They were used in experiments at a weight of 20–30 g. Solid tumours were grown by injecting 2–5 $\times 10^6$ cells into the gastrocnemius muscle of the hind limb. Cells were obtained from a previous xenograft passage by sterile disaggregation of one or more tumours and preparation of a single cell suspension using $1\,mg\,ml^{-1}$ bacterial neutral protease (Sigma, Type IX). The experiments described in this paper were all carried out using xenograft-passage 2–4 from the *in vitro* cell line. Tumour growth was assessed by measuring two leg diameters at right angles using a perspex gauge in association with a calibration curve of leg diameter *versus* tumour weight (Twentyman *et al.*, 1979). IFN-γ administration was commenced when individual tumours reached 200–300 mg and tumours were then measured twice weekly as was mouse body weight. The time for each individual tumour to attain 4× its weight at the onset of treatment was then assessed. IFN-γ was administered either by the i.p. or the s.c. route at a dose of $4 \times 10^5$ IU per mouse per day in a volume of 0.1 ml per mouse per day. It was diluted to the appropriate concentration in storage buffer or saline, aliquoted and refrozen prior to use. In some experiments, aliquots were thawed immediately before administration. In others, sufficient IFN for 2–3 days treatment was thawed and stored at 4°C. Appropriate solvent controls were included in all experiments.

### Results

### *Effects of IFNs on cell growth*

In a series of initial experiments, the effect of IFN-α and IFN-γ at a dose of $10^3\,IU\,ml^{-1}$ on the growth of various cell types was studied. The data are shown in Table II. It may be seen that both types of IFN had some effect on the growth of the human cells, but there was no clear effect on the mouse cells (EMT6/Ca/VJAC and RIF-1). Among the human cells, POC and COR-L23 were the most sensitive (especially to IFN-γ) whereas NCI-H69 was relatively resistant. Growth curves for POC and NCI-H69 in the presence of $10^3\,IU\,ml^{-1}$ of IFN-α or IFN-γ are shown in Figure 1. These data confirm the relative sensitivity of POC compared with NCI-H69 and also the greater effect of IFN-γ compared with IFN-α. Growth curves (not shown) for COR-L23 confirmed this latter point. We also obtained dose-response data for IFN-α and for our 2 batches of IFN-γ on POC and COR-L23. Data for COR-L23 are shown in Figure 2. It may be seen that the 2 batches of IFN-γ were of similar potency and the IFN-α was less effective. A similar conclusion was drawn from the data for POC (not shown). On the basis of these data it was decided to concentrate attention in subsequent experiments upon the effects of IFN-γ on POC and COR-L23.

More complete dose-response data for the effect of IFN-γ on these cells are shown in Figure 3. The curves for both cell lines fall rapidly between 0 and $500\,IU\,ml^{-1}$ of IFN-γ with little further effect with increasing dose.

We also carried out experiments to study the effect on cell growth of IFN-γ replenished daily *versus* a single addition at the onset of the experiments. The data are shown in Table III. In

**Table II** Effect of IFN-α and IFN-γ on the growth of cell lines.

| | *Exp. 1* *cells/flask* (× $10^5$) | | | *Exp. 2* *cells/flask* (× $10^5$) | | |
|---|---|---|---|---|---|---|
| | *CONT* | *IFN-α* | *IFN-γ* | *CONT* | *IFN-α* | *IFN-γ* |
| BEN | 6.8 | 5.2 | 3.6 | 10.4 | 12.0 | 8.0 |
| MOR | 4.4 | 2.6 | 3.3 | 8.2 | 4.6 | 4.9 |
| POC | 4.6 | 2.5 | 1.4 | 7.8 | 4.2 | 3.7 |
| NCI-H69 | 6.1 | 4.2 | 4.9 | 4.0 | 3.8 | 3.1 |
| COR-L23 | 3.7 | 2.4 | 0.9 | 7.4 | 5.1 | 1.6 |
| EMT6/Ca/VJAC | 24.5 | 25.3 | 28.3 | 37.0 | 35.0 | 42.0 |
| RIF-1 | 24.5 | 22.0 | 24.4 | 11.2 | 9.7 | 8.2 |

Notes:

(a) Figures are counts of phase-contrast viable cells from single flasks.
(b) IFNs added at $10^3\,IU\,ml^{-1}$ on day 0 and unchanged throughout.
(c) Experiment 1 – all flasks set up at $10^5$ cells on day 0
Final counts: EMT6/Ca/VJAC; RIF-1 – day 3
NCI-H69; BEN; MOR; COR-L23 – day 6
POC – day 8.
(d) Experiment 2 – all flasks set up at $10^5$ cells on day 0 except NCI-H69 = $2 \times 10^5$ cells on day 0.
Final counts: EMT6/Ca/VJAC; RIF-1 – day 3
NCI-H69 – day 6
BEN; MOR; COR-L23 – day 8
POC – day 9.

**Figure 1** Effect on the growth of NCI-H69 cells (a) and POC cells (b) of IFN-α or IFN-γ added at $10^3$ IU ml$^{-1}$. (○) Control; (■) IFN-α; (▲) IFN-γ.

**Figure 2** Effect of different doses of IFN-α (■) or two different batches of IFN-γ (▲, △) on the growth of COR-L23 cells. Flasks were inoculated with $10^5$ cells on day 0 and IFN added immediately. Cells/flask were counted on day 9.

both POC experiments there was a greater effect of IFN-γ when replenished daily. For COR-L23, however, there did not appear to be a great difference between the effects of the two regimes.

A recent report (Bakhanashvili *et al.*, 1983) has shown that cells can be more sensitive to the inhibitory effects of IFN when grown in low serum concentrations. In order to test whether the high sensitivity of COR-L23 was due to the fact that we grow this line in 2.5% serum rather than the 10% used for the other lines, we carried out a comparative study using high and low serum concentrations. The results are shown in Table IV. It may be seen that the effect of IFN-γ on COR-L23 was not dependent upon the medium used.

*Flow cytometry studies*

As the cells in COR-L23 have an extremely heterogeneous distribution of DNA content per nucleus, we used line POC to examine the effect of IFN-γ on cell cycle distribution. The data from 2 such experiments are shown in Table V. In the first

**Table III** Comparison of the effects of a single addition of IFN-$\gamma$ to growth medium versus daily renewal of IFN-$\gamma$.

| *Experiment* | *Cell line* | *Dose of IFN-γ IU ml$^{-1}$* | *Cells/flask (× $10^5$) single IFN-γ* | *daily IFN-γ* | *Note* |
|---|---|---|---|---|---|
| 1 | POC | 0 | 14.8 | 12.7 | 1 |
| | | $10^3$ | 4.8 | 1.7 | |
| 2 | POC | 0 | 18.0 | 20.2 | 2 |
| | | 200 | 17.5 | 10.9 | |
| | | $10^3$ | 12.0 | 10.2 | |
| 3 | COR-L23 | 0 | 6.0 | 8.3 | 3 |
| | | $10^3$ | 3.6 | 3.7 | |
| 4 | COR-L23 | 0 | 14.0 | 11.0 | 4 |
| | | $10^3$ | 5.8 | 6.3 | |

1. Set up at $3 \times 10^5$/flask, counted at day 7.
2. Set up at $2 \times 10^5$/flask, counted at day 8.
3. Set up at $10^5$/flask, counted at day 6.
4. Set up at $10^5$/flask, counted at day 6.

**Figure 3** Effect of different doses of IFN-$\gamma$ on the growth of POC (●) and COR-L23 (○) cells. For POC the flasks were inoculated with $2 \times 10^5$ cells on day 0 and counted on day 9. For COR-L23 cells, the flasks were inoculated with $10^5$ cells on day 0 and counted on day 7. IFN was added to flasks immediately after the cells.

experiment (A) IFN-$\gamma$ was added to the flasks immediately after the cells on day 0. In experiment B, flasks were inoculated on day 0 but then allowed to grow for 3 days before addition of the IFN to ensure that the results were not influenced by the disaggregation procedure. In each experiment an

**Table IV** Effect of IFN-$\gamma$ on the growth of COR-L23 in two different growth media.

| *Medium* | *IFN-γ dose IU ml$^{-1}$* | *Cells/flask (× $10^5$)* |
|---|---|---|
| HITES + 2.5% FCS | 0 | 9.9 |
| | 500 | 7.2 |
| | $2 \times 10^3$ | 3.4 |
| RPMI + 10% FCS | 0 | 9.5 |
| | 500 | 6.9 |
| | $2 \times 10^3$ | 2.9 |

Flasks set up at $10^5$ cells on day 0, counted on day 6.

effect of the IFN-$\gamma$ on cell growth was observed but there was no clear effect upon cell cycle distribution.

*Effect upon spheroid growth*

In order to compare the effects of IFN-$\gamma$ on cells in suspension with that on multicellular tumour spheroids we carried out the following procedure. Cells of the POC line were inoculated into flasks and allowed to grow into spheroids of 200–400 $\mu$m diameter (as described in **Materials and methods**). At this time, some of the spheroids were disaggregated and flasks inoculated with $2 \times 10^5$ cells per flask. In addition, individual spheroids were placed into single wells on 24 well multidishes and measured daily. IFN-$\gamma$ ($10^3$ IU ml$^{-1}$) was added to flasks or wells at the onset of this experiment and either left throughout the experiment or changed daily. The results of these experiments are shown in Table VI. In experiment A there was a relatively small effect on the cells growing in flasks when the IFN-$\gamma$ was left unchanged and this regime had no effect on

**Table V** Effect of IFN-γ on cell growth and cell cycle distribution of POC cells.

| *Exp.* | *Day* | *IFN-γ IU ml$^{-1}$* | *Cells/flask × $10^5$* | *% of cells in G1* | *S* | *G2* |
|---|---|---|---|---|---|---|
| A[a] | 0 | — | 10.0 | 52.8 | 38.2 | 9.0 |
| | 2 | 0 | 12.6 | 47.9 | 40.1 | 12.0 |
| | | $10^3$ | 8.8 | 46.8 | 36.5 | 16.7 |
| | 3 | 0 | 19.6 | 50.0 | 41.4 | 8.6 |
| | | $10^3$ | 12.4 | 49.0 | 36.4 | 14.5 |
| | 6 | 0 | 38.8 | 54.7 | 37.3 | 8.1 |
| | | $10^3$ | 26.4 | 53.3 | 36.9 | 9.8 |
| B[b] | 0 | — | 10.0 | 44.3 | 42.9 | 12.7 |
| | 4 | 0 | 15.8 | 44.1 | 44.9 | 11.0 |
| | | $10^3$ | 14.5 | 41.5 | 42.9 | 15.6 |
| | | $5 \times 10^3$ | 14.2 | 43.7 | 42.9 | 13.3 |
| | 5 | 0 | 27.6 | | | |
| | | $10^3$ | 22.6 | | | |
| | | $5 \times 10^3$ | 22.2 | | | |
| | 6 | 0 | 31.0 | 49.0 | 36.1 | 14.9 |
| | | $10^3$ | 24.5 | 49.1 | 38.5 | 12.3 |
| | | $5 \times 10^3$ | 24.4 | 48.8 | 34.2 | 17.0 |

[a]In experiment A, flasks were inoculated with $10^6$ cells/flask on day 0 and IFN-γ was added at the same time.

[b]In experiment B, flasks were inoculated with $5 \times 10^5$ cells/flask on day 0, IFN-γ was added on day 3.

**Table VI** Effect of IFN-γ on spheroid growth compared with cell multiplication in POC cells.

| *Exp.* | *Daily medium change* | *IFN-γ dose IU/ml* | *Cells/flask at day 7 (× $10^6$)[a]* | *Mean spheroid diameter day 0 (μm)[b]* | *Mean spheroid diameter day 7 (μm)* | *Increase in diameter (μm)* |
|---|---|---|---|---|---|---|
| A | NO | 0 | 2.21 | 445 | 735 | 290 |
| | NO | $10^3$ | 1.69 | 430 | 740 | 310 |
| | YES | 0 | 1.25 | 425 | 730 | 305 |
| | YES | $10^3$ | 0.62 | 430 | 690 | 260 |
| B | NO | 0 | 1.56 | 330 | 710 | 380 |
| | NO | $10^3$ | 1.75 | 315 | 735 | 420 |
| | YES | 0 | 0.95 | 240 | 635 | 385 |
| | YES | $10^3$ | 0.70 | 315 | 630 | 315 |
| C | NO | 0 | 1.68 | 448(31) | 885(28) | 437(41) |
| | NO | $10^3$ | 1.02 | 383(21) | 763(62) | 383(65) |
| | YES | 0 | 1.16 | 439(32) | 801(26) | 362(41) |
| | YES | $10^3$ | 0.49 | 418(26) | 768(41) | 350(49) |

[a]2 flasks per point – values given are mean counts.

[b]8–12 spheroids per treatment group.

In experiments A and B, data on individual spheroid diameters were not stored and hence s.e. not given.

In experiment C, figures in parentheses indicate 2 s.e. of the mean.

**Table VII** Effect of IFN-$\gamma$ on growth on human cell lines as tumours in nude mice.

| | | | *Time to reach 4 × treatment size (days)* | | |
|---|---|---|---|---|---|
| *Exp.* | *Cell line* | *Route of IFN admin*[a] | *Buffer control* | *IFN-$\gamma$ Batch 2* | *IFN-$\gamma$ Batch 1* |
| A | POC | i.p. | 7.6 (6.8–8.2) $n=15$ | 7.3 (6.2–8.5) $n=14$ | — |
| B | NCI-H69 | i.p. | 9.1 (7.8–10.6) $n=6$ | 9.4 (7.6–11.8) $n=6$ | — |
| C | COR-L23 | s.c. | 8.2 (6.4–10.6) $n=7$ | 8.3 (7.1–9.7) $n=6$ | — |
| D | COR-L23 | s.c. | 6.9 (5.9–8.1) $n=6$ | 6.8 (5.5–8.2) $n=6$ | 6.3 (5.2–7.6) $n=6$ |

[a]Dose $=4\times10^5$ i.u. per mouse per day (equivalent to $1.6\times10^7\,\mathrm{IU\,kg^{-1}\,day^{-1}}$ or $4.8\times10^7\,\mathrm{IU\,m^{-2}\,day^{-1}}$).

Values of time in days are geometric means of groups of $n$ mice with 2 s.e. limits in parentheses.

spheroid growth. A larger effect was seen in flasks with daily medium change and a small inhibition of spheroid growth was also observed. In experiment B, a single administration of IFN-$\gamma$ produced no inhibition (in fact a small increase) in cells per flask and also in spheroid growth. Again the daily medium change regime was rather more effective in both systems. In experiment C, results in flasks are similar to those of experiment A, whereas spheroid growth was suppressed more for single than for repeated administration of IFN. The two standard error limits on mean spheroid diameter values in experiment C, however, indicate that the differences are not statistically significant and the same almost certainly applies to the equivalent data for experiments A and B. It would appear, therefore, that major differences seen in cells per flask (experiments A and C) for repeated administration of IFN-$\gamma$ do not result in equally large suppression of spheroid growth. This may indicate a reduced sensitivity of cells in large 3-dimensional aggregates or a failure of IFN to penetrate into the spheroids.

### *Effect* in vivo

The data from 4 experiments carried out using human lung cell lines grown as tumours in nude mice are shown in Table VI. There was no significant effect of IFN-$\gamma$ in any of the experiments. No weight loss was seen in any of the groups of mice, neither were there any other visible signs of toxicity with the exception of one death due to haematoma at the site of s.c. injection.

### Discussion

The results presented in this paper indicate that both IFN-$\alpha$ and IFN-$\gamma$ are able to cause a partial suppression of the growth of some human lung cancer cell lines. In a direct comparison at a dose of $10^3\,\mathrm{IU\,ml^{-1}}$, the effect of IFN-$\gamma$ was generally greater than that of IFN-$\alpha$ on the two most sensitive lines (POC and COR-L23) and this comparison was confirmed over a wide range of doses. The effect of IFN-$\gamma$ was related to dose over the region 0–$10^4\,\mathrm{i.u.\,ml^{-1}}$ but the major part of the effect occurred below a dose of $500\,\mathrm{IU\,ml^{-1}}$. The major effect of IFN-$\gamma$ on cell growth curves was greatest at early times with later growth becoming almost parallel to control. These data on cell sensitivity to IFN-$\alpha$ and IFN-$\gamma$ may be compared with data from the literature for the effect of IFN-$\alpha$ on other cell lines (reviewed by Stewart, 1979). The Daudi line of human lymphoblastoid cells is particularly sensitive (Adams *et al.*, 1975; Gewart *et al.*, 1981) (i.e. effects seen at less than $10\,\mathrm{IU\,ml^{-1}}$) whereas others are resistant to several thousand units. Our lines POC and COR-L23 may therefore be regarded as of intermediate sensitivity whereas the lines showing little or no response to $10^3\,\mathrm{IU\,ml^{-1}}$ may be regarded as resistant.

Our flow cytometry studies did not reveal any major perturbations in the cell cycle distribution of cells growing in the presence of IFN-$\gamma$. Although some workers have found that IFNs do not cause major perturbations in cell cycle distribution (Killander *et al.*, 1976; Balkwill *et al.*, 1978), a very wide diversity of effects of IFNs on cell cycle kinetics and phase distribution have, however, been reported by others (Watenabe *et al.*, 1978; Lundgren *et al.*, 1979; Creasey *et al.*, 1980; Lundblad & Lundgren, 1981; Roos *et al.*, 1984). In the study by Lundblad and Lundgren (1981) the effects of IFN-$\alpha$ and IFN-$\beta$ on a glioma cell line in exponential growth were studied using flow cytometry. The proportion of cells with DNA contents corresponding to the S phase of the cell cycle was markedly increased in IFN treated cells. The authors combined their flow cytometry data with data obtained using labelling techniques to conclude that IFN was causing cessation of DNA synthesis before normal replication was complete. A number of human haemopoietic cell lines and fresh leukaemic cells were studied by Roos *et al.* (1984). They found that the main effect of IFN-$\alpha$ was to cause a block in the Go-$G_1$ phase of the cell cycle, although the Burkitt's lymphoma line, Namalwa, showed decreased progress through S without any Go/$G_1$ accumulation, thereby leading to an increased S phase fraction judged by flow cytometry. Our flow cytometry data are difficult to interpret in detail in the absence of labelling studies to measure the rate of DNA synthesis. Clearly, however, major perturbations in cell cycle distribution which have been reported by others do not occur in the POC cell line treated with IFN-$\gamma$. The growth inhibiting effects occurring at the time when flow cytometry was performed must therefore be dependent upon change in the rate of progress of cells around the cycle rather than major blocking at a specific point.

The negative results for the effect of $4\times10^5$ i.u. day$^{-1}$ of IFN-$\gamma$ on tumours growing in nude mice are disappointing. It has been shown by Balkwill *et al.* (1982) that IFN-$\alpha$ is able to slow the growth of a human breast carcinoma xenograft if given in daily doses of $2\times10^5$ IU to nude mice. The same total weekly dose (i.e. $1.4\times10^6$ IU) either as a single dose or as 3 doses per week was much less effective. These doses were chosen as being approximately equivalent to the doses (based on IU m$^{-2}$) which are achievable in man. A similar dose regime for IFN-$\alpha$ was used by Clutterbuck *et al.* (1983) to treat immune-deprived mice bearing xenografts of human lung adenocarcinoma, melanoma or acute myeloid leukaemia. In these experiments, IFN administration was carried out daily from the day before tumour implant for 14 days. No effect was seen on the growth of any of the tumours. The conclusion reached by Balkwill *et al.* (1982) from their data was that the positive response observed was likely to be due to a direct effect on the human cells rather than a host effect upon the nude mice used. If this is so, it appears that the psotive response to IFN-$\gamma$ which we have observed for COR-L23 and POC cells *in vitro* does not directly predict for a similar response in xenografted tumours. It may be, however, that this negative result is due to pharmacokinetic reasons. No detectable anti-viral activity was detected in patients' serum following i.m. injection of partially pure IFN-$\gamma$ and non-glycosylated recombinant IFN-$\gamma$ appeared to be poorly absorbed in the squirrel monkey after i.m. administration (Gutterman *et al.*, 1984). We were unable, however, to carry out repeated i.v. injections into nude mice and thus are unable to directly compare routes of administration.

It is far from clear to what extent clinical response to IFNs is dependent upon direct cytotoxicity or cytostasis in the tumour cell population. In addition, a detailed knowledge of the pharmacokinetics of IFN-$\gamma$ in both the mouse and in man will be necessary for a direct comparison of *in vitro, in vivo* and clinical results. The implications of our data for the likely effect of IFN-$\gamma$ in clinical trials of lung cancer therapy must therefore remain uncertain.

We thank Mr S. Chambers and Dr J.V. Watson for carrying out flow cytometric analysis of treated populations of cells and Mrs Jane Donaldson for her help with xenograft experiments.

### References

ADAMS, A., STRANDER, H. & CANTELL, K. (1975). Sensitivity of Epstein–Barr virus transformed human lymphoid cell lines to interferon. *J. Gen. Virol.*, **28**, 207.

BAKHANASHVILI, M., WRESCHNER, D.H. & SALZBERG, S. (1983). Specific antigrowth effect of interferon on mouse cells transformed by murine sarcoma virus. *Cancer Res.*, **43**, 1289.

BALKWILL, F.R., WATLING, D. & TAYLOR-PAPADIMITRIOU, J. (1978). Inhibition by lymphoblastoid interferon of growth of cells derived from the human breast. *Int. J. Cancer*, **22**, 258.

BALKWILL, F.R., MOODIE, E.M., FREEDMAN, V. & FANTES, K.H. (1982). Human interferon inhibits the growth of established human breast tumours in the nude mouse. *Int. J. Cancer*, **30**, 231.

CANTELL, K. & HIRVONEN, S. (1977). Preparation of human leucocyte interferon for clinical use. *Tex. Rep. Biol. Med.*, **35**, 138.

CARNEY, D.N., BUNN, P.A., Jr., GAZDAR, A.F., PAGAN, J.A. & MINNA, J.D. (1981). Selective growth in serum-free hormone-supplemented medium of tumor cells obtained by biopsy from patients with small cell carcinoma of the lung. *Proc. Natl Acad. Sci.*, **78**, 3185.

CLUTTERBUCK, R.D., MILLAR, J.L. & ALEXANDER, P. (1983). Failure of high doses of $\alpha$ interferon to affect the growth of human carcinoma, melanoma and myeloid leukaemia xenografts. *Br. J. Cancer*, **48**, 445.

CREASEY, A.A., BARTHOLOMEN, J.C. & MERIGEN, T.C. (1980). The importance of Go in the site of action of interferons in the cell cycle. *Exp. Cell Res.*, **134**, 155.

GEWERT, D.R., SHAH, S. & CLEMENS, M.J. (1981). Inhibition of cell division by interferons. Changes in the transport and intracellular metabolism of thymidine in human lymphoblastoid (Daudi) cells. *Eur. J. Biochem.*, **116**, 487.

GUTTERMAN, J.U., ROSENBLUN, M.G., RIOS, A. FRITSCHE, H.A. & QUESADA, J.R. (1984). Pharmacokinetic study of partially pure $\gamma$-interferon in cancer patients. *Cancer Res.*, **44**, 4164.

JONES, D.H., BLEEHEN, N.M., SLATER, A.J., GEORGE, P.J.M., WALKER, J.R. & DIXON, A.K. (1983). Human lymphoblastoid interferon in the treatment of small cell lung cancer. *Br. J. Cancer*, **47**, 361.

KILLANDER, D., LINDAHL, P., LUNDIN, L., LEARY, P. & GRESSER, I. (1976). Relationship between the enhanced expression of histocompatibility antigens on interferon-treated L1210 cells and their position in the cell cycle. *Eur. J. Immunol.*, **6**, 56.

LUNDBLAD, D. & LUNDGREN, E. (1981). Block of a glioma cell line in S by interferon. *Int. J. Cancer*, **27**, 749.

LUNDGREN, E., LARSON, I., MIORNER, H. & STRANNEGARD, O. (1979). Effect of leucocyte and fibroblast interferon on events in the fibroblast cell cycle. *J. Gen. Virol.*, **42**, 589.

PAUKER, K., CANTELL, K. & HENLE, W. (1962). Quantitative studies on viral interference in suspended L-cells. III Effects of interfering viruses and interferon on the growth rate of cells. *Virology*, **17**, 324.

ROOS, G., LEONDERSON, T. & LUNDGREN, E. (1984). Interferon-induced cell cycle changes in human haemopoietic cell lines and fresh leukemic cells. *Cancer Res.*, **44**, 2358.

STEWART, II, W.E. (1974). Distinct molecular species of interferons. *Virology*, **61**, 80.

STEWART II, W.E. (1979). In *The Interferon System*, New York: Springer Verlag, p. 241.

STOOPLER, M.B., KROWN, S.E., GRALLA, R.J., CUNNINGHAM-RANDLES, S., STEWART, W.E. & DETTGEN, H.F. (1980). Phase II trial of human leukocyte interferon in non-small cell lung cancer. In: *Abstracts of II World Conference on Lung Cancer*. (Eds Hansen & Dombernowsky), Amsterdam: Excerpta Medica, p. 221.

TAYLOR, I.W. (1980). A rapid single step staining technique for DNA analysis by flow microflourim *J. Histochem. Cytochem.*, **28**, 1021.

TAYLOR-PAPADIMITRIOU, J. (1980). Effects of interferons on cell growth and function. In *Interferon*, **2**, p. 13. (Ed. Gressor) Academic Press: New York.

TWENTYMAN, P.R. (1980). Response to chemotherapy of EMT6 spheroids as measured by growth delay and cell survival. *Br. J. Cancer*, **42**, 297.

TWENTYMAN, P.R. (1982). Growth delay in small EMT6 spheroids induced by cytotoxic drugs and its modification by misionidazole pretreatment under hypoxic conditions. *Br. J. Cancer*, **45**, 565.

TWENTYMAN, P.R., KALLMAN, R.F. & BROWN, J.M. (1979). The effect of time between X-irradiation and chemotherapy on the growth of three solid mouse tumours – 1. Adriamycin. *Int. J. Radiat. Oncol. Biol. Phys.*, **5**, 1255.

WATANABE, Y. & SOKAWA, Y. (1978). Effect of interferon on the cell cycle of BALB/C 3T3 cells. *J. Gen. Virol.*, **41**, 411.

WATSON, J.V. (1980). Enzyme kinetic studies in cell populations using fluorogenic substrates and flow cytometric techniques. *Cytometry*, **1**, 143.

WATSON, J.V., CHAMBERS, S.H. & SMITH, P.J. (1985). A pragmatic approach to the analysis of DNA histograms with a definable $G_1$ peak. (Submitted to *Cytometry*).